# Developing a Scalable Annotation Method for Large Datasets That Enhances Alarms With Actionability Data to Increase Informativeness: Mixed Methods Approach

**DOI:** 10.2196/65961

**Published:** 2025-05-05

**Authors:** Sophie Anne Inès Klopfenstein, Anne Rike Flint, Patrick Heeren, Mona Prendke, Amin Chaoui, Thomas Ocker, Jonas Chromik, Bert Arnrich, Felix Balzer, Akira-Sebastian Poncette

**Affiliations:** 1 Institute of Medical Informatics Charité – Universitätsmedizin Berlin, corporate member of Freie Universität Berlin and Humboldt Universität zu Berlin Berlin Germany; 2 Core Facility Digital Medicine and Interoperability Berlin Institute of Health at Charité – Universitätsmedizin Berlin Berlin Germany; 3 Department of Anesthesiology and Intensive Care Medicine Charité – Universitätsmedizin Berlin, corporate member of Freie Universität Berlin and Humboldt Universität zu Berlin Berlin Germany; 4 Digital Health - Connected Healthcare Hasso-Plattner-Institute University of Potsdam Potsdam Germany; 5 Einstein Center Digital Future Berlin Germany

**Keywords:** alarm management, alarm fatigue, alarm informativeness, patient monitoring, dataset annotation, intensive care unit, transdisciplinary research, machine learning, technological innovation, patient-centered care, digital health

## Abstract

**Background:**

Alarm fatigue, a multifactorial desensitization of staff to alarms, can harm both patients and health care staff in intensive care units (ICUs), especially due to false and nonactionable alarms. Increasing amounts of routinely collected alarm and ICU patient data are paving the way for training machine learning (ML) models that may help reduce the number of nonactionable alarms, potentially increasing alarm informativeness and reducing alarm fatigue. At present, however, there is no publicly available dataset or process that routinely collects information on alarm actionability (ie, whether an alarm triggers a medical intervention or not), which is a key feature for developing meaningful ML models for alarm management. Furthermore, case-based manual annotation is too slow and resource intensive for large amounts of data.

**Objective:**

We propose a scalable method to annotate patient monitoring alarms associated with patient-related variables regarding their actionability. While the method is aimed to be used primarily in our institution, other clinicians, scientists, and industry stakeholders could reuse it to build their own datasets.

**Methods:**

The interdisciplinary research team followed a mixed methods approach to develop the annotation method, using data-driven, qualitative, and empirical strategies. The iterative process consisted of six steps: (1) defining alarm terms; (2) reaching a consensus on an annotation concept and documentation structure; (3) defining physiological alarm conditions, related medical interventions, and time windows to assess; (4) developing mapping tables; (5) creating the annotation rule set; and (6) evaluating the generated content. All decisions were made based on feasibility criteria, clinical relevance, occurrence frequency, data availability and quantity, structure, and storage mode. The annotation guideline development process was preceded by the analysis of the institution’s data and systems, the evaluation of device manuals, and a systematic literature review.

**Results:**

In a multidisciplinary consensus-based approach, we defined preprocessing steps and a rule-based annotation method to classify alarms as either actionable or nonactionable based on data from the patient data management system. We have presented our experience in developing the annotation method and provided the generated resources. The method focuses on respiratory and medication management interventions and includes 8 general rules in a tabular format that are accompanied by graphical examples. Mapping tables enable handling unstructured information and are referenced in the annotation rule set.

**Conclusions:**

Our annotation method will enable a large number of alarms to be labeled semiautomatically, retrospectively, and quickly, and will provide information on their actionability based on further patient data. This will make it possible to generate annotated datasets for ML models in alarm management and alarm fatigue research. We believe that our annotation method and the resources provided are universal enough and could be used by others to prepare data for future ML projects, even beyond the topic of alarms.

## Introduction

Vital sign monitoring and alarm systems in intensive care units (ICUs) aim to inform caregivers about the health status of patients and about adverse events. Alarms can be either clinically actionable or nonactionable as defined by the International Electrotechnical Commission (IEC) in an amendment of the second edition of its norm IEC 60601-1-8:2006/AMD2:2020 (sections 3.44 and 3.45, respectively), a norm defining general requirements, tests, and guidance for alarm systems in medical electrical equipment and systems, with an alarm being considered actionable if an intervention is expected within a time window defined by the alarm priority to prevent or counteract physiological deterioration [1]. In fact, less than 23% of alarms in ICUs are actionable [2-5], and ICU staff reported high rates of alarms that do not have consequences and disturb their work [6]. At our institution, nurses and physicians interact with monitoring systems and are involved in alarm management tasks [7]. Interventions after an alarm are made in consultation with or by physicians or based on their orders. However, the literature has shown that nonactionable alarms can promote alarm fatigue, a multifactorial desensitization of personnel that is associated with a “delayed, inadequate, inappropriate, or absent [response]” to an alarm [1], and can ultimately harm both patients and health care professionals [8-12] as they are considered to impact staff performance and patient safety [1].

While there was no valid metric to quantify alarm fatigue until the recent publication of the standardized and validated “Charité Alarm Fatigue Questionnaire” (CAFQa) [13,14], Winters et al [15] hypothesized that reducing the total number of alarms and specifically nonactionable alarms might alleviate alarm fatigue. However, until now, many alarm research projects have focused on classifying alarms as either technically true or false (due to artifacts or not having a valid triggering event), without further analyzing their clinical actionability [16]. The CAFQa captures alarm fatigue across the dimensions of alarm stress and alarm coping, enabling the evaluation of the psychophysiological effects of excessive alarms and also strategies for managing alarms [13,14]. Yet, it has not been used in interventional studies to assess alarm fatigue in ICU staff or the impact of any approach aiming to alleviate alarm fatigue. It is unknown whether reducing the number of nonactionable alarms and in general improving the informativeness, often measured as the positive predictive value of alarms, can cause a decrease in alarm fatigue and alarm response problems [15,17].

Datasets containing alarms classified based on their actionability are essential to develop holistic IT approaches that aim to tackle alarm fatigue [16]. Information related to alarm events is mostly not included in publicly available ICU databases, such as the eICU Collaborative Research Database [18], HiRID [19], and AmsterdamUMCdb [20], or is only in the form of alarm thresholds, such as in the Medical Information Mart for Intensive Care (MIMIC)-IV [21,22]. This might be due to the fact that alarm data are commonly not used for health care provision and often remain in local systems for quality management processes. These data are stored in vendor proprietary formats and require extensive preprocessing to be useful [23]. Data-driven applications, including machine learning (ML) algorithms, depend on real-world health data and shared health care datasets. To ensure data diversity, reproducibility, generalizability, and sharing, they ideally encompass standards and sustainable approaches to ultimately provide insights into health care delivery and improve patient care and outcomes [24-28]. As a prerequisite for any supervised ML framework, these data need to be annotated (ie, indicating the clinical actionability of alarms). As such, the algorithm learns from the patterns of the annotated input data in combination with other relevant data points and produces accurate results [29]. However, this annotation step is frequently the main bottleneck because it is complex [30], is time and resource consuming [31,32], and requires domain expertise.

We propose a rule-based annotation method to enrich alarm data triggered by physiological alarm conditions (PACs) and captured by a patient monitoring system (ie, electrocardiogram, invasive blood pressure [BP] device, or pulse oximeter) by providing information about their actionability based on further patient data related to respiratory and medication management interventions. This will serve as a basis to semiautomatically annotate large amounts of alarm data. By sharing this reproducible and scalable annotation method, including preprocessing recommendations, we aim to enable clinical and research institutions to label their own alarm data in order to aid in analyzing their ICU alarm situation for quality management and to facilitate projects involving ML in academia and industry for new patient monitoring systems.

## Methods

### Ethical Considerations

The ethics committee of Charité – Universitätsmedizin Berlin approved all protocols of this study (ethics vote number: EA1/127/18; name of the ethics committee: Ethikausschuss der Charité – Universitätsmedizin Berlin am Campus Charité Mitte; Chairperson: Prof Dr med R Morgenstern). Due to the setting and scope of the study, Charité’s data protection department and the abovementioned ethics committee waived the requirement to obtain informed consent.

### Data Sources and Materials

Our study focused on creating annotation guidelines based on patient and alarm data from the ICUs of a large German university hospital. These data are stored in different systems.

The patient data management system (PDMS) COPRA (version 6) allows for the documentation of patient data, such as observations and vital signs, medications, diagnoses, procedures, and further information related to the hospital stay. Some information is also stored in the hospital information system (HIS) i.s.h.med (eg, laboratory results) and might be transmitted additionally to the PDMS. Some ventilation settings (eg, positive end-expiratory pressure) are stored in the ventilators and transmitted automatically to the PDMS, while others require manual documentation (eg, airway devices [ADs]).

Alarm logs are present in the Philips IntelliVue patient monitoring system (MX800 software version M.00.03; MMS X2 software version H.15.41-M.00.04). It includes bedside monitors, client monitors integrating data from 2 to 3 patient rooms, and a central station. By default, it measures oxygen saturation (SpO_2_), BP (invasive or noninvasive), heart rate (HR), and temperature. In our study, we focused on a subset of patient alarms triggered by the electrocardiogram, invasive BP device, or pulse oximeter.

We created a project database structured according to Giesa et al [33], which included data from 35,004 patients and 40,865 distinct stays. Patient and alarm data arising from 19 units (ICUs, postanesthesia care units, recovery rooms, and operation rooms) between August 2019 and June 2021 were collected in the PDMS, HIS, and alarm logs. To develop and test our annotation method, we focused solely on data from ICUs related to 7163 unique patients (cases and unique patient counts per ICU ward in Table S1 in Multimedia Appendix 1; age and gender distribution with patient counts in Table S2 in Multimedia Appendix 1). We counted 13,473,594 alarm starts in ICUs, and the alarm signals associated with the chosen PACs accounted for 50.72% of these alarm starts. Only the alarm logs of ICU M101I were complete over the whole time span. M101I is a surgical ICU, with its alarms representing 17.93% of the total number of alarms in our database.

As alarm logs are only stored locally in Philips’ central station for 90 days, these were regularly extracted using a USB stick; extensively processed by applying R scripts [34], which are partly based on R scripts by Poncette et al [35]; and imported into the database.

### Study Design and Research Team

We chose a mixed methods approach, blending quantitative strategies, data-driven as well as qualitative evidence-based strategies, and empirical strategies to inform the development process of our annotation guideline. The iterative consensus-based process integrated adapted methods from requirements engineering and design thinking, which are 2 complementary frameworks [36,37]. Requirements engineering facilitates the definition, maintenance, and validation of requirements for the development of systems or tools [38]. Design thinking fosters a creative process that rapidly generates and prioritizes effective solutions. It incorporates environmental and user perspectives, either directly (eg, through watching, examination, surveying, etc) or indirectly (eg, through empathizing, assumption, etc) [39,40], and has already been used in different health care settings and projects [41-45], including intensive care medicine [46]. Even though both frameworks were developed in different contexts and propose different tools, they both aim to understand and solve a problem, especially in the context of developing software. While design thinking is user-centered and explores the needs and sociotechnical, operational, and usability aspects (including limiting factors), requirements engineering enables detailing of the properties of a product and testing these in the development process. The produced contents of both frameworks are partially overlapping or complementary [36,37]. All results are presented in tabular form and as figures. For the figures, we integrated the color blindness accessibility color palette proposed by Wong [47].

The core team consisted of 2 physicians experienced in anesthesiology and intensive care medicine (1 junior doctor with additional expertise in interoperability and standardization, and 1 senior doctor having completed his training in anesthesiology), 3 data scientists, and 2 medical students (1 with a bioinformatics degree). In addition, 6 intensive care medicine specialists (2 senior consultants having completed their training in anesthesiology and holding an additional qualification in intensive care medicine and 4 senior residents in anesthesiology, with 3 of these involved in data science projects) and 1 IT expert supported the process by participating in workshops and interviews. The different levels of expertise and experience made it possible to include different perspectives and develop a pragmatic yet clinically focused method, which is reusable as it incorporates international standards and norms.

### Preliminary Work

Prior to the development of an annotation method, we performed a literature review to find relevant articles about alarm annotation projects or methods in the ICU and surgical setting (Web of Science and Embase; Figure S1 in Multimedia Appendix 2 shows the selection process according to the PRISMA [Preferred Reporting Items for Systematic Reviews and Meta-Analyses] 2020 statement [48]). In parallel, we collected and analyzed internal information (eg, data sources, database structures, data formats and flows, systems, device settings, or working processes) and the instruction manuals of devices. We interviewed 7 different domain experts from intensive care medicine and IT using open-ended and specific questions (Table S1 in Multimedia Appendix 3). All findings were summarized, presented, and discussed in regular research meetings.

### Development of the Alarm Annotation Guidelines

We iteratively conducted 6 steps to create and refine our annotation method. Throughout the process, we prioritized and consolidated ideas, and made decisions based on feasibility criteria (time, personnel, and technical resources), clinical relevance, frequency of occurrence, data availability and quantity, structure, and storage mode.

### Definitions of Alarm Terms

The terms annotation and labeling of alarms are used synonymously in this manuscript. Alarm terms are taken from the IEC norm 60601-1-8:2006 and its amendments from 2012 and 2020. Monitoring systems use alarm signals, commonly simply called alarms, to convey the presence of an alarm condition [49]. Alarm conditions represent situations that monitoring systems classify as potentially or actually dangerous and require staff to intervene or at least be aware [50]. Alarm conditions are triggered by a violation of alarm limits, which can be numerical, nonnumerical, or algorithmic thresholds. Despite an alarm condition, the measured value might still be in the physiological range. Alarm conditions can emanate from patient-related and equipment-related variables, which are named PACs and technical alarm conditions, respectively. For the annotation, we focused on PACs and considered related alarms singularly, although several alarms can occur simultaneously. We disregarded alarm trends or patterns. Technical alarms (eg, alarms resulting from device disconnection) were excluded.

Adapting the definition of “clinically actionable” according to IEC 60601-1-8:2006/AMD2:2020 (section 3.44) [1], an alarm is “actionable” if health care staff react to it by performing an intervention to counteract physiological deterioration in a certain time window. We do not consider, for example, assessing a patient, changing alarm limits, and repositioning a misplaced device, as interventions to prevent harm [1]. At this stage, we also do not distinguish between the different alarm priorities.

### Agreement on a General Annotation Concept and Definition of a Documentation Structure

Based on the findings of the preliminary work and domain expertise, the core team identified and discussed potential annotation methods before deciding on a rule-based annotation method using PDMS data and alarm logs. One team member proposed ideas for the structures of the requirement specifications, mappings, annotation rules, and tables for the annotation output, and oversaw the management and updates of the documents. The entire team reviewed and approved these resources.

### Selecting PACs of Interest, Related Medical Interventions, and Time Windows to Assess Alarm Actionability

We identified relevant data elements in the alarm logs providing insights about the patient (identified using the unit and bed number), the timing of alarm events (using timestamps), and the PAC (via the vital sign type and the threshold violation). For our annotation method, we focused on PACs related to SpO_2_, invasive BP, and HR. Each alarm was evaluated individually.

One medical doctor examined which interventions were usually documented (shortly) after alarm events using data from the PDMS and HIS. Based on this retrospective analysis as well as their medical and clinical knowledge and experience, 3 medical doctors independently listed medical interventions that are usually performed to counteract a physiological deterioration for each selected PAC. The selection of interventions was guided by the most common reasons and etiologies that lead to alarm conditions and signals. The lists were merged, discussed, and consolidated. Interventions were excluded if they were technical (eg, change of an electrode), were poorly documented or not documented in a timely manner (eg, positioning), were documented using free text, were of a diagnostic nature, or were not performed directly after the first alarm (eg, blood transfusion). The proposed list of interventions is not meant to be exhaustive. In the following text, we concentrate on 2 types of interventions: respiratory and medication management interventions.

Respiratory management interventions summarize interventions related to the increase in set ventilation parameters and change in the ventilation situation (change in airway management or respiratory support therapy [RST], including the presence or absence of oxygen delivery). Airway management is concerned with the manipulation of ADs and gives information about the invasiveness of respiratory support. RSTs consider combinations of ventilation devices (VDs) and ventilation modes (VMs). In our database, oxygen therapy is also documented as a combination of a VD and VM despite not involving ventilation. ADs, VDs, and VMs are stored as strings, while ventilation parameters are delivered as numerical values in our PDMS. We focused on escalations of respiratory management after alarms.

Medication management interventions include all types of changes in administration aiming to counteract physiological deterioration (increase or decrease in dosage and administration start or stop).

To assess the alarm actionability, we set a time window to consider after the alarm start. Its definition was based on clinical expertise, technical knowledge (eg, documentation processes, technical factors, such as data transmission and storage frequencies for every variable and ICU, and imprecisions arising from system interfaces), data visualizations of performed interventions in relation to alarm timestamps, and count of interventions performed as a result of different time windows.

To later test the generated annotation rule set, we retrieved all variables depicting the PACs, related alarm information, and previously defined medical interventions from the source systems and stored these in a project database as explained in the Data Sources and Materials subsection.

### Development of Mappings to Describe Respiratory and Medication Management Interventions

To process and compare unstructured data, we created mappings for both groups of interventions: respiratory and medication management. We retrieved, indexed, and mapped entries from the PDMS manually. The mappings augment the information content of the (unstructured) data and make it easily interpretable for both machines and people without a medical background.

#### Development of Respiratory Management Mappings

There is no standard definition or guideline to describe escalations of respiratory management (neither airway management nor respiratory support). We used clinical expertise, information from ISO19223:2019 [51], and instruction manuals of ventilators to define a total of 18 categories for ADs and 7 categories for RSTs, and to perform the actual mappings that can be used to determine if an escalation was performed.

To indicate the invasiveness of the therapy, we assigned a level to each AD category: increasing AD levels indicate an escalation of airway management, with level 1 being “no AD” documented and level 9 being a “tracheal cannula” or “endotracheal tube.” Unclear AD entries were mapped to several suitable categories. One example would be the entry “Maske” that could represent an oxygen mask or a nasal or full-face mask for continuous positive airway pressure therapy. However, for entries having multiple mappings, we specified the level to use to annotate the alarms.

We defined RST categories based on the intended use of the ventilators and ignored their adjuncts (eg, tube compensation). RST categories represent breathing therapy and VMs. We attributed each RST category a level. VDs and VMs are always stored in combination in our PDMS. We mapped every VD-VM combination that we extracted to an RST category and level. In rare cases, when a VD-VM combination could be linked to several RST categories, the AD was included in the decision process. A higher RST level is associated with a more severe respiratory therapy, as patients either get more respiratory support or need a “controlled” VM. We assigned each VD-VM combination an AD category that is suitable to conduct a therapy with this specific combination. When the AD was not documented, we defined a “default” invasiveness based on clinical judgment. In this case, we added “blank/no AD” to the VD-VM combination to store this information.

An additional table specifies which ventilation parameters can be set in the context of a chosen RST. Questions related to specific ADs, VDs, and VMs were answered during one-on-one meetings. The generated mappings were discussed with intensive care experts, and a random sample of the mapping content was cross-validated in a workshop. The ventilation parameters table was validated by a senior physician.

#### Development of Medication Management Mappings

Depending on the PAC, health care personnel need to manage different medications to prevent harm. For alarms triggered by a specific vital sign value decrease, we considered (1) active ingredients that would counteract the deterioration when administered or increased in dosage, and (2) active ingredients that would counteract the deterioration when stopped or reduced in dosage. Analogously, for alarms triggered by a value increase, we chose active ingredients that would lead to a decrease or stabilization of the value and mapped these to the 2 medication intervention types.

The medication mapping was based on information retrieved from the internal hospital medication database, medical experience, and knowledge. We looked for relevant medication information in the PDMS database, including names (eg, database-specific names, trade names, and generic names), routes and techniques of administration, and the hospital’s custom drug identifier (DrugID). Each active ingredient can have several DrugIDs depending, for example, on the route of administration or drug concentration. Therefore, a mapping of relevant DrugIDs to selected active ingredients was assembled. All substances were mapped to concepts of the Systematized Nomenclature of Medicine–Clinical Terms. We defined 8 categories for routes and 2 for techniques of administration, and harmonized these.

In our hospital, medication interventions are linked to an order identifier (orderID) combining one or several substances. For orderIDs with more than one substance (“mixtures”), we analyzed all unique substance combinations in the patient data to identify relevant mixtures for the annotation.

One medical doctor selected potentially relevant active ingredients for each PAC and type of medication management intervention and performed the mapping. The result was presented in one-on-one meetings to 3 intensive care specialists asked to assess the accuracy and coverage of common real-world scenarios, leading to the first consolidation based on their judgment. Subsequently, we evaluated the mapping by analyzing actual patient data. We queried medication interventions for each PAC, 2 alarm criticality levels, and 3 different time windows (5, 10, and 15 minutes), and ordered the resulting lists by descending count. Two medical experts checked if the listed active ingredients (after having set a cumulative percentual cutoff of 70%) were clinically relevant in the context of each PAC [52]. These findings were used to revise the list of chosen active ingredients and finalize the medication mapping.

### Definition of the Annotation Rule Set

Based on the previously selected alarm and patient variables and mappings, the team of medical experts defined rules to compare one or several specific variables before an alarm (or at the time of an alarm) and after an alarm within a specified postalarm time window. If specific conditions are met in a determined time window, depicting an intervention, the alarm is actionable; otherwise, it is not actionable. The technical experts helped synthesize and generalize the rules. This served as preliminary work for the development and implementation of scripts.

### Evaluation of the Generated Content

We started programming R and Python scripts to test the implementation of the annotation rules and mappings, and applied the test scripts to a subset of alarm and patient data. Using bed names and timestamps, we linked the logs to patients in the PDMS. We created graphics to simulate and represent common and complex scenarios. Complex scenarios relate, among others, to situations where interventions occur in parallel or sequentially within the defined time window, situations where medication management interventions include the same substance as another ongoing continuous administration, or situations where known common documentation errors need mitigation, such as by implementing checking loops or delays. We also performed regular data queries to augment and refine the generated contents (eg, add entries to the mappings or implement further checks to assess the alarm actionability). Initial annotation results were cross-checked by assessing related patient data, both in the PDMS frontend and backend. All contents were discussed regarding conciseness, intelligibility, and usability, from a medical and data science point of view.

## Results

### Overview

We first present the factors prompting the development of our annotation method before sharing the generated resources.

### Preliminary Work

We identified 20 studies containing 23 associated alarm annotation reports in our literature review [4,30-32,53-71] (PRISMA [48] flow diagram: Figure S1 in Multimedia Appendix 2) and summarized the findings (Table S1 and Table S2 in Multimedia Appendix 2). Although the annotation methods and outcomes of interest differed across all studies, most of the reports specified definitions or protocols prior to the annotation process to classify the alarms. The size of the annotated dataset ranged from 20 [69] to 12,671 [59,65] alarms, with an average of 4183 annotated alarms. Aspects regarding the scalability of the annotation method were not reported.

We identified and evaluated 4 potential annotation methods for our project (Table 1), blending medical and data science perspectives. These included manual approaches, such as annotating alarms in person in an ICU and analyzing information in the PDMS frontend, and semiautomatic approaches, such as rule-based systems and ML algorithms. Medical users (both interviewees and team members) emphasized the need for highly precise annotations (despite limited time resources) that capture the reasons for alarm actionability. Data scientists, on the other hand, focused on maximizing the number of annotated alarms and aimed for scalability.

**Table 1:** Advantages and disadvantages of different alarm annotation methods.

Method	Time-stamped annotation in person in the ICU^a^	Annotation through visualization of records in the PDMS^b^ frontend or based on video material	Semiautomatic rule-based annotation	Automatic annotation
Method description	Medical experts annotate in real-time and at the bedside the clinical interventions performed after patient alarms in the ICU	Medical experts investigate patient data using the PDMS frontend or video recordings (showing, for example, the patient and their surroundings), and assess the available data close to alarm timestamps using medical knowledge and experience	Development of rule-based logic and annotation guidelines using routine clinical patient data and medical knowledge; Implementation of this logic with computer scripts	Automatic annotation based on ML^c^ algorithms
Time perspective	Prospective	Retrospective	Retrospective	Retrospective
Data sources	Information on alarms and bedside tasks recorded by annotators	Alarm logs from the monitoring system; all patient information recorded in the PDMS or on video	Alarm logs from the monitoring system; selected patient features from the PDMS/HIS^d^, health data lake, or project-specific database	Alarm logs from the monitoring system; selected patient features from the PDMS/HIS, health data lake, or project-specific database
Personal resources for annotation	Persons with a medical background	Persons with a medical background	To develop the guidelines: persons with a medical background; to implement the logic: persons with data science or IT skills	Persons with data science or IT skills; no medical skills needed
Time resources for annotation	High	High	Medium	Low
Data size	Limited by the prospective collection setting; greater amount with increasing data collection duration or number of annotators	Limited by the annotation setting; greater amount with increasing annotation period or number of annotators	Potentially all alarm logs that include alarm types considered by the annotation guidelines	Potentially all alarm logs
Data quality	High; “clinical annotations are generally accepted as an alternative [to a gold standard]” [53]	Medium to high, depending on, for example, if (1) rules or criteria have been defined prior to the annotation, (2) annotation is conducted by independent mappers, and (3) there are adjudication rounds	Medium	Medium
Scalability	Very limited	Limited	Yes	Yes
Limitations	Administrative and organizational efforts; considerable time and personal resources	Considerable time and personal resources; method is dependent on the precise documentation of information in the PDMS or detailed video recordings	Extensive data preprocessing and wrangling, especially when the PDMS is not based on a standardized model; method is dependent on the precise documentation of information in the PDMS	Supervised learning requires an existing algorithm that has been trained and tested on an already labeled dataset and preprocessed data; method is dependent on the precise documentation of information in the PDMS
Summary	Most precise data annotation method out of the 4 presented here, but very resource intensive and not scalable	Less precise data annotation than if annotated in real-time at the bedside, but less resource intensive and slightly more scalable	Depending on the rules and how well they depict the reality, this method can achieve a precise data annotation; scalable due to automatic annotation once extensive data preprocessing is done	In theory, this method has the potential to be the best annotation method, as it is scalable, is less resource intensive, and can annotate large amounts of data once an algorithm has been developed, but it is dependent on a pre-existing labeled dataset or pretrained algorithm

^a^ICU: intensive care unit.

^b^PDMS: patient data management system.

^c^ML: machine learning.

^d^HIS: hospital information system.

### Alarm Annotation Method

#### General Annotation Concept and Documentation Structure

We agreed on a semiautomatic annotation method after having analyzed in depth our internal data systems and the collected information about different processes (Table S2 in Multimedia Appendix 3). It consisted of the development of a detailed deterministic and machine-readable rule set, which was designed with algorithmic application in mind.

The rule set has been specified using tables accompanied by explanatory texts with considerations to be applied for concrete use cases, mappings, tables for the annotation output, and exemplary visualizations. The entire mappings are available on Zenodo [72]. Examples are included in the supplementary materials. Our proposal to structure the annotation output (Table S1 and Table S2 in Multimedia Appendix 4) indicates the reason it is annotated as actionable for each alarm. The guidelines contain no individual patient data, despite being generated based on analysis and extraction of alarm logs, data from the PDMS, and data from the HIS. For this publication, we present a generic version of the method.

### Selected PACs, Medical Interventions, and Time Windows

**Figure 1:**
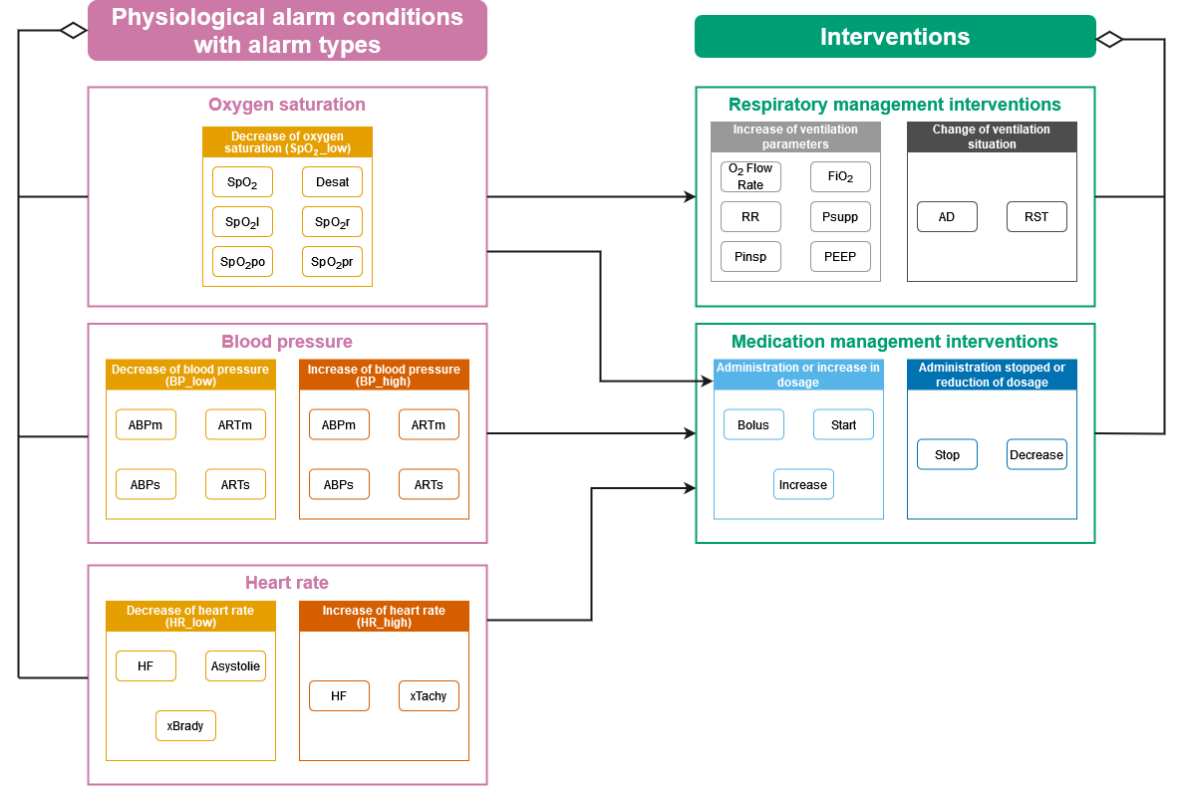
Overview of the selected physiological alarm conditions (PACs) and interventions related to our annotation method. A patient can trigger zero, one, or many alarms. Each alarm can be followed by zero, one, or multiple interventions. The figure displays the alarm names as they are generated by our monitoring system. ABPm/ARTm: mean arterial blood pressure; ABPs/ARTs: systolic arterial blood pressure; AD: airway device; Asystolie: (German alarm name for) asystole; Desat: desaturation; FiO2: fraction of inspired oxygen; HF: (German alarm name for) heartrate; PEEP: positive end-expiratory pressure; Pinsp: inspiratory pressure; Psupp: pressure support; RR: respiratory rate; RST: respiratory support therapy; SpO_2_: oxygen saturation; SpO_2_l: oxygen saturation left side; SpO_2_po: oxygen saturation post ductal; SpO_2_pr: oxygen saturation pre ductal; SpO_2_r: oxygen saturation right side; xBrady: extreme bradycardia; xTachy: extreme tachycardia.

We chose PACs and interventions to be evaluated in our annotation method (Figure 1). In total, 14 different alarm types from the alarm logs were clustered into 5 PACs. Thirteen actions in airway and medication management are summarized under “Interventions.” The annotation method was based on the evaluation of PACs and interventions in relation to each other. We assessed the actionability of the related alarms by seeing if the defined interventions were conducted in a timely manner. In our method, we considered airway and medication management interventions for SpO_2_ alarms and only assessed medication management interventions for BP and HR alarms. Alarm signals can be co-occurring, reflecting different problems or being related to the same problem and reflecting compensatory mechanisms of body functions. For now, we annotated each alarm individually. For respiratory management interventions, we considered a time window of 30 minutes after the alarm, and for medication management interventions, we considered 15 minutes.

### Generated Mappings for Respiratory and Medication Management Interventions

To ease the comparison of specific variables and enable the annotation, we performed mappings [72] for both intervention groups.

#### Respiratory Management–Related Mappings

We retrieved a total of 1033 unique strings representing ADs, including errors such as diverse spellings, missing entries, or blanks. We mapped 49 unclear entries to 2 suitable AD categories, 20 to 3 categories, and 5 to 4 categories, leading to a total of 1137 combinations of strings and AD categories. For the alarm annotation, we ignored entries mapped to AD level 0 as these did not correspond to ADs, were VDs or systems delivering therapeutic substances, or were documentation errors (Table S3 in Multimedia Appendix 4 shows AD categories with examples and mapping numbers).

We extracted a total of 80 VDs and 264 VMs for mapping to RSTs. This resulted in 3461 unique combinations of VD-VM-AD categories. Our current mapping covers 519,248 theoretical VD-VM-AD entry combinations.

#### Medication Management–Related Mappings

We listed 47 active ingredients and identified 840 associated unique DrugIDs. The annotation rules take the routes and techniques of administration into account. We subsumed 15 unique routes into 8 categories and mapped these to the techniques “bolus” and “continuous intravenous administration.” Table S4 in Multimedia Appendix 4 presents the general structure of the medication mapping with the numbers of considered products and examples.

### Concrete Annotation Rule Set

Linking alarms to interventions is based on the integration of the information of PACs, PDMS variables, mappings, timings, and time windows. As several changes in ventilation parameter settings might occur after an alarm, we considered the peak value of a variable in the defined postalarm time window. Prior to the annotation, values not compatible with life should be removed, for example, by using a table adapted from the MIMIC-III project [73,74] defining physiological ranges, ranges compatible with life, and minimum and maximum acceptable outliers. Regular planned interventions (eg, planned medication administration or prophylactic continuous positive airway pressure to prevent pneumonia) should also be excluded based on the screening of related prescriptions made in advance as they are not executed in reaction to an alarm.

#### Annotation Rules for Respiratory Management Interventions

We focused on the change in ventilation parameter settings and the ventilation situation (Table 2). The rules are as follows:

1. Consider the following time windows and values: (1) the AD and RST levels at the time of the alarm start (use ventilation mapping and AD mapping to determine the levels) [72]; (2) the last value of a ventilation parameter before the alarm; and (3) the maximum values after the alarm in a specified postalarm time window of 30 minutes measured after the alarm start.
2. Check if the patient has an AD: An AD is considered inserted if an AD type is documented with a timestamp; an AD is considered removed if there is a related removal timestamp or if a new AD type is documented.
3. Check if the VD is in standby mode: For set ventilator parameters (except for oxygen flow rate in the context of oxygen therapy) and RST rules (except for RST spontaneous breathing and oxygen therapy), if the VD is in standby mode, set the values of the set ventilation parameter and RST level to 0; otherwise, consider the last value. Note that RST level 0 is introduced for implementation purposes.
4. Check if the ventilation parameter can be set in the context of this RST: For set ventilator parameter rules, additionally check the RST at the time of the parameter setting. In case of incompatibility, set the parameter value to 0; otherwise, consider the last value.

For each RST, we have listed suitable ventilation parameters in Table 3.

**Table 2:** Annotation rules for respiratory management interventions (physiological alarm condition: SpO_2__low).

Rule	Logic	Condition 1	Logic	Condition 2	Logic	Condition 3
Change of AD^a^	IF	Last (AD level)	<	Max (AD level)	AND	No removal logged
Change of RST^b^	IF	Last (RST level)	<	Max (RST level)	AND	No standby^c^
Increase in a set numerical ventilation parameter (oxygen flow rate, fraction of inspired oxygen, set rate, inspiratory pressure, pressure support, or positive end-expiratory pressure)	IF	Last (set parameter X)	<	Max (set parameter X)	AND	Parameter can be set in the present RST level

^a^AD: airway device.

^b^RST: respiratory support therapy.

^c^Standby is allowed in case of spontaneous breathing or during oxygen therapy.

**Table 3:** Compatibility table of ventilation parameters and respiratory support therapies.

RST^a^ level	Ventilation parameter								
	Set the O_2_ flow rate (on a flowmeter)	Set the O_2_ flow rate (not on a flowmeter)	Set FiO_2_^b^	Set PEEP^c^	Set Psupp^d^	Set Pinsp^e^	Set RR^f^		
1^g^	No	No	No	No	No	No	No		
2	Yes	No	No	No	No	No	No		
3	No	No	Yes	Yes	No	No	No		
4	No	Yes	Yes	No	No	No	No		
5	No	Yes	Yes	Yes	Yes	No	No		
6	No	Yes	Yes	Yes	Yes	Yes	Yes		
7	No	Yes	Yes	Yes	No	Yes	Yes		

^a^RST: respiratory support therapy.

^b^FiO_2_: fraction of inspired oxygen.

^c^PEEP: positive end-expiratory pressure.

^d^Psupp: pressure support.

^e^Pinsp: inspiratory pressure.

^f^RR: respiratory rate.

^g^Level 1 is formally not a respiratory support therapy.

#### Annotation Rules for Medication Management Interventions

Procedures related to the management of drug administration can impact the vital signs we focused on. We considered 2 general medication intervention types: “administration or increase in dosage” and “administration stopped or reduction in dosage” (Table 4). The rules are as follows:

1. Consider the following time windows and values: A postalarm time window of 15 minutes measured after an alarm starts, and the rate at the time of the alarm start for continuous administration.
2. Check the active ingredients: The medication mapping [72] specifies the active ingredients to consider depending on the medication intervention, PAC, and administration technique. All rules focus on 1 active ingredient at a time. For mixtures (combinations of two or more active ingredients), check in the mixture mapping if the combination is relevant for the annotation.
3. Check the administration technique: If the start time is equal to the end time, the technique is bolus; otherwise, the technique is continuous. In the case of continuous administration, we compared the rates (these are documented in the PDMS or need to be calculated). The rates can only be compared if they share the same unit (if not, they need to be converted) and the same concentration. In the case of a different concentration, the administered doses per time need to be used for comparison.
4. Check if a fluid is used as a therapy or as a diluent or carrier (only for active ingredients classified as “fluid for intravenous administration”): If the amount (or rate) is <500 mL (per hour), fluid is a carrier; otherwise, it is a therapy.

**Table 4:** Annotation rules for medication management interventions.

Rule		Logic	Technique	Logic	Condition 1	Logic	Condition 2	Logic	Condition 3	Logic	Condition 4
**Administration or increase in dosage (PAC^a^: all)**											
	Bolus	IF	Bolus	AND	Administration within the time window	—^b^	—	—	—	—	—
	Start	IF	Continuous	AND	New administration starts within the time window	AND	No (previous) administration ends in the previous 5 minutes	AND	No further administration of the same active ingredient running in parallel	ELSE	Check for increase
	Increase	IF	Continuous	AND	New administration starts within the time window	AND	Previous administration ends within the previous 5 minutes	AND	New rate > previous rate^c^	—	—
**Administration stopped or reduction in dosage (PAC: all except SpO_2__low)**											
	Stop	IF	Continuous	AND	Administration ends within the time window	AND	No new administration starts following 5 minutes	AND	No further administration of the same active ingredient running in parallel	ELSE	Check for decrease
	Decrease	IF	Continuous	AND	Previous administration ends within the time window	AND	New administration starts following 5 minutes	AND	Previous rate > new rate^c^	—	—

^a^PAC: physiological alarm condition.

^b^Not applicable.

^c^In case of a comparison of 2 continuous administrations with different DrugIDs and concentrations (eg, propofol 10 mg/mL and propofol 20 mg/mL), consider the doses per time instead of the rates.

### Evaluation of the Generated Content

**Figure 2:**
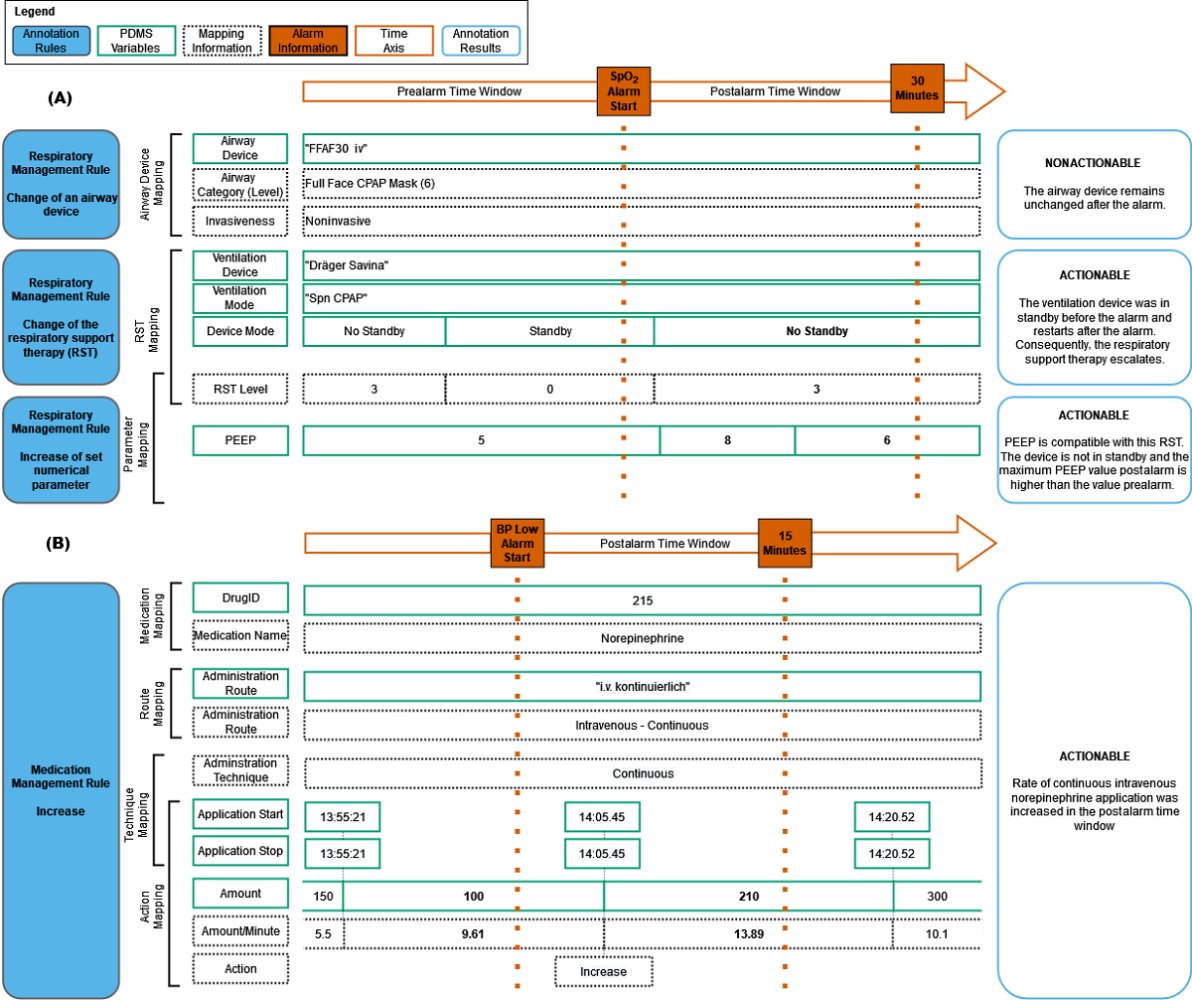
Visualization of annotation rules with example data and annotation results. (A) Respiratory management rules. This panel depicts which patient data management system (PDMS) variables are required by the 3 respiratory management rules, how they interact with the different mappings, and which time windows are considered. (B) Medication increase rule. This panel depicts the PDMS variables required to map the medication name, application route and technique, and medication action. BP: blood pressure; CPAP: continuous positive airway pressure; PEEP: positive end-expiratory pressure; RST: respiratory support therapy.

We used visual examples to simulate and define the annotation output for different scenarios (Figure 2A and 2B; Figures S1 to S4 in Multimedia Appendix 4). Annotation rules that seem trivial at first (eg, checking if a ventilation parameter is increased after an alarm) involve knowing the type of RST before and after the alarm, the device mode (standby or not), if this parameter can be set in the context of the specific RST, and the set value before and after the alarm (last and maximum value, respectively). For example, a PDMS data entry for positive end-expiratory pressure is faulty for oxygen therapy. In 9.0% of documented AD changes, the removal of the preceding AD was missing. To account for known cases, in which the new AD was documented shortly before the removal of the previous one, we added a tolerance of 1 minute, which reduced the rate to 7.21% of AD changes. Of the 230,711 changes of VDs or VMs (not related to RST 1 or 2), 53,875 were incorrectly documented as performed while the VD was in standby. For a subset of 49,072 of these changes, standby was subsequently deactivated, with 50% of deactivations occurring within the first 7 minutes and 75% occurring within the first 20 minutes. We considered alarms as nonactionable if changes were documented while the standby mode was active. Checking loops and short delays helped to ensure that the PDMS data accurately reflected the ICU situation.

Although we first used medication names in the annotation, we noticed that it was necessary to use the hospital’s custom medication identifiers, as medication naming conventions were not consistent in our PDMS and included structured and free-text entries. We also noticed that nearly 60% of the medication application timestamps were rounded down to the nearest 5 minutes, caused by the standard PDMS user interface input dialog. If not accounted for, this would cause a significant number of false negatives, leading to an underestimation of the number of actionable alarms.

We normalized the rule tables to reduce redundancies and make them machine readable.

Medical experts controlled if the code of the first developed scripts accurately reflected the medical content of annotation guidelines. Inaccuracies were mainly due to prerequisites not being formulated precisely in the guidelines, misunderstanding of rules, or “wrong” variables or timestamps being used in the code. These findings led to further refinement of the annotation guidelines. Data scientists also evaluated the runtime required for annotation and tested various code optimization and parallelization strategies. They successfully processed over 1000 alarms per minute, with potential for further scaling through the addition of hardware resources.

## Discussion

### Principal Findings

Alarm fatigue and improper management of clinical alarm systems are still highly relevant topics, as underlined by the 6th National Patient Safety Goals of the Joint Commission’s hospital program for 2024 [75]. As publicly available ICU datasets lack alarm information, we developed a method to classify large amounts of alarms regarding their clinical actionability using a defined annotation rule set combining alarm data and patient health data, to enable the creation of such an alarm dataset.

The ICU environment is particularly interesting for ML projects due to large data volumes produced by the multitude of devices and monitoring systems, and the necessity of rapid decisions when treating critically ill patients. While some routinely collected data can be used directly (eg, vital signs), others need complex preprocessing based on contextual data or clinical judgment to make sense of the information. This is, for example, the case for the alarm metric “clinically actionable” [76] or to understand the concrete ventilation situation. However, as soon as annotations move toward big data, manual annotation is not feasible. Semiautomatic annotation is faster and less resource-intensive than manual annotation at the bedside once the data are extracted and preprocessed and the rule-based algorithm is implemented [77].

In the context of alarm management and research, there is still no consensus on a gold standard method of how to annotate alarms [53]. Past studies used different methods, definitions, and wordings to classify alarms or assess their actionability, as our literature review showed. However, using widely accepted definitions and standardized nomenclature is crucial to ensure the comparability of findings. Our annotation method is explainable, reproducible, and scalable. It incorporates definitions from ISO and IEC norms, fostering future data reusability and interoperability. The guidelines (instructions with illustrative examples) ensure coherence, explain how to deal with unusual cases [78-80], and could serve as a basis for future code development and implementation of the annotation rules. In accordance with the principle “start small, iterate fast” [81], we opted for a modular layout and incremental development, with regular testing phases to find out how IT developers understand and transpose the guidelines, identify complex cases and inconsistencies, and ultimately refine the guidelines. The modular layout will also facilitate future adaptation and addition of rules or underlying mappings, as new data points (eg, further VDs or active ingredients) might need to be added.

Comprehensive knowledge and understanding regarding the ICU environment, the involved persons, the processes, and the data were essential for our project. We involved clinicians and IT experts in the development process from the beginning, thus including different points of view, expertise, and experience levels, and aimed to create reusable content. Their participation was crucial in the iterative analysis of the ICU ecosystem, evaluation of actual data, testing phases, and discussions. We recommend allocating enough time and resources to these activities, as they are crucial for making informed decisions. Routine health data are stored for documentation purposes rather than for analysis tasks that would benefit from structured machine-readable formats. We identified challenges (Table S2 in Multimedia Appendix 3) that were associated with complex time- and resource-intensive measures (eg, decision-making, data processing and deduplication, plausibility checks, and mappings).

All generated resources are mostly generic and reusable, potentially time-saving, and helpful for a larger audience and different stakeholders. They could be used to create new datasets or enrich existing ones, such as MIMIC-III and MIMIC-IV, with lower annotation efforts. MIMIC-III and MIMIC-IV are both freely available databases including data from patients admitted to critical care units in the Beth Israel Deaconess Medical Center (Boston, MA, USA). MIMIC-IV contains data from about 200,000 patients admitted to the emergency department and 65,000 admitted to an ICU between 2008 and 2022 [21,22]. While alarm data are sparse in both datasets, alarm thresholds are available, and alarm events could be extracted [82]. By enriching existing datasets, research possibilities would be extended [77] without researchers being confronted with legal uncertainties around anonymization and sharing of a newly created health dataset. Concerning alarms, clinicians could get detailed alarm summaries, including information about alarm actionability as a new metric, fostering active alarm management, evidence-based decision-making, and interprofessional discussions [76]. Patient monitoring systems aim to keep patients safe, but nonactionable alarms are regarded as prejudicial to staff performance and patient safety [1]. Research and industry projects could use our method to create annotated alarm datasets to evaluate the effects of differing alarm actionability rates on outcomes, such as length of stay and mortality, or to train ML models discriminating and predicting actionable and nonactionable alarms (improving the low positive predictive value and therefore informativeness of current patient monitoring systems) and potentially guide clinicians in their decisions by moving from predictive to actionable artificial intelligence [83]. In the clinical routine, such algorithms should be trained on datasets including labeled alarms and contextual information (patient data before and after an alarm such as conditions, diagnostic and therapeutic interventions, co-occurring or occurring alarms, etc) to enable prediction. They could assist clinicians, for example, by informing them how likely an intervention might be required after an alarm related to a PAC, and enable trials investigating if the reduction or suppression of nonactionable alarms can lead to reduced alarm fatigue using the validated tool CAFQa [13,14]. As both nurses and physicians interact with patient monitoring systems and are involved in alarm management [7], analyses assessing if the impact and perception of such algorithms in practice differ between both professional groups should follow. Our current annotation guidelines only focus on few potential interventions after an alarm. We are aware that other interventions might be possible and should be considered in the future to increase the precision of the annotated alarm datasets and improve subsequent analyses and algorithms.

As our mappings provide insights about patients’ ventilation and medication situation independently of alarm events, they are suitable for health care projects beyond the alarm research context. They enable, for example, to easily summarize how many patients were ventilated invasively and which kind of medication is often administered in a particular department or situation. They could be used to build more granular features for ML compared to current common approaches (eg, binary ventilation feature: vented yes or no? [84]) or for medical controlling and quality insurance purposes.

### Limitations

Our current annotation guidelines allow a partial analysis of the alarm situation in the ICU and cannot be used on their own for prediction purposes or decision-making. They are based on analyses of retrospective data and expert opinions to annotate a subset of alarms regarding their actionability based on chosen clinical interventions. The underlying assumptions, proposed rules, and data used to develop our annotation process are subject to limitations.

Annotation methods should be seen as living processes, as continuous refinement and iterative development will be necessary to capture additional scenarios that are not yet anticipated. The sample of involved experts and the pandemic situation at the time of the study influenced the decision to develop a semiautomatic rule-based annotation method. The methodical framework that guided our study was not evaluated against another approach. In our annotation method, alarms are considered nonactionable if they do not fulfill the criteria of a defined rule. Thus, an alarm might be annotated as “nonactionable” because no rule was defined to capture a specific intervention or because the relevant data were missing or imprecise despite being actionable. Currently, we can only presume associations between alarms and interventions because alarms and interventions were stored in 2 different systems. The interventions that are listed in the system are not marked as happening as a direct response to an alarm: For example, when a change in a ventilator parameter is logged, there is no link to the SpO_2_ drop and saturation alarm preceding this change. Interventions that led to an alarm being annotated as “actionable” might not have been related to it. By annotating each alarm individually, we ignored whether there were any co-occurring alarms. Co-occurring alarms could be related to a single problem (when multiple alarms are triggered because of compensatory changes) or indicate entirely different problems.

We did not derive our rules based on prospectively collected data or validate these in a clinical setting. We relied on retrospective data and especially on documentation interfaces, processes, and data accuracy. Due to the management of several seriously ill patients, stress, working routine, and documentation burden, health care professionals might not document interventions in a timely or precise manner. Additionally, the storage frequency, data precision, and diverse timestamps for the same variable might also influence which conclusions can be drawn from the annotations. We therefore prioritized partially structured data, primarily quantitative data, data automatically transmitted from the respective devices to the PDMS, and data that were manually documented in a timely manner. Free-text notes were ignored. The list of alarm types and associated interventions is not meant to be exhaustive yet and only enables an initial analysis of the ICU alarm situation, with a potential underestimation of the rate of nonactionable alarms. Not all etiologies of alarm conditions are covered by the chosen interventions. Additionally, the annotation is partially based on complex mappings and data structuring. Further evaluation and expansion should follow. Our current respiratory management mappings are “static” and ignore dynamic parameters that might have an impact on the real-time classification of some VMs. Mappings should be modulated based on adjuncts. Concerning medication management, we focused on medications that have been added to the underlying PDMS database. Training, institutional policies, and patient comorbidities might influence the choice of medication. Our selection is based on medical experience and retrospective data analysis. The medication mapping is not exhaustive and might need adaptation for other institutions or countries, for which the relevant medication groups developed in our mapping can serve as orientation. Researchers need to factor in the previously described challenges and limitations when preparing and annotating their data by implementing, for example, checking loops or delays. Performing a prospective data annotation at the bedside would enable us to better understand previously identified challenges, validate and refine our current logic, and prioritize new rules for integration.

Alarm log data were regularly extracted from Philips’ central stations of up to 13 ICUs as the data were not transferred automatically to our HIS or data lake. With new data coming in, the local monitor system buffer fills up, causing older log entries to be overwritten and resulting in a storage duration of approximately 90 days. This short storage duration is a rather common limitation in bedside monitoring systems, and it is up to the institutions to decide if they want to transfer and store the logs. We could not prevent missing alarm logs as the data collection required additional organization and volunteers, and started at different points of time. These technical and organizational challenges might underline the fact that alarm-related research and analyses are often not prioritized yet. The alarm scripts developed to process the logs might be reused, especially for the log transformation and mapping parts. However, this requires alarm log structures and content to be consistent across manufacturers. In our opinion, easier access, better storage solutions, and standardized logs would ease alarm research and management.

Like commonly used threshold-based monitoring systems, our annotation method neither considers alarm and vital sign trends nor alarm combinations or trains. However, threshold-based alarms might not accurately reflect the physiological state of a patient at a given moment. Integrating vital signs or other parameters in the annotation logic could help to assess whether an intervention really happened. If the vital sign that triggered the alarm stabilizes or returns to normal in a timely manner after the intervention, the likelihood that the alarm and intervention are related would be higher. Other researchers have tried to reduce the number and enhance the information content of alarms, for example, by considering patient motion [85] or categorical laboratory test results [86] in the alarm evaluation or creating sequences composed of, for example, alarm combinations, values of vital signs, laboratory results, and further parameters to predict conditions and events [87,88]. The use and visualization of parameter trends and sequences are not limited to alarm research [89-91]. Exploring and integrating the mentioned approaches in our annotation might improve the accuracy of the labels. Our annotation method focuses on enriching alarms with actionability information, but other features could also be used to label alarms and further increase alarm informativeness. Besides the addition of new information (eg, actionability) and decrease of nonactionable alarms, future patient monitoring systems might also need to refactor existing alarm categories and rethink ways to represent and convey information to further increase alarm informativeness [17,92].

Subsequent trials need to evaluate whether our annotation method and future systems and algorithms based on datasets labeled using our method are clinically accurate and help address alarm fatigue.

### Conclusions

Our annotation method opens new clinical and research opportunities in the alarm research field and beyond. Stakeholders from different domains, such as clinicians, researchers, and alarm system manufacturers, could make use of our annotation rules and mappings, as they are generic enough to be reused after alignment with their own hospital database structures for analyses and model development. Existing ICU databases could be enriched with new annotations and metrics. Thereby, our annotation method can ultimately enhance and enable in-depth data analyses and ML possibilities in ICUs and beyond, especially regarding the alarm situation, and support research aiming to counteract alarm fatigue. The next step is to make the datasets, including the annotated alarms, openly available so that new research projects can develop the next generation of monitoring systems.

## Appendix

Multimedia Appendix 1 Demographics.

Multimedia Appendix 2 Literature review on alarm annotation projects and methods in the intensive care unit and surgical setting.

Multimedia Appendix 3 Questions asked to experts during one-on-one meetings and workshops, and challenges identified through exploratory analyses of the data sources, structures, and entries from our institution.

Multimedia Appendix 4 Proposed annotation output structure and examples illustrating the preprocessing steps and annotation algorithm.

## Abbreviations

AD: airway device
BP: blood pressure
CAFQa: Charité Alarm Fatigue Questionnaire
DrugID: drug identifier
HIS: hospital information system
HR: heart rate
ICU: intensive care unit
IEC: International Electrotechnical Commission
MIMIC: Medical Information Mart for Intensive Care
ML: machine learning
PAC: physiological alarm condition
PDMS: patient data management system
PRISMA: Preferred Reporting Items for Systematic Reviews and Meta-Analyses
RST: respiratory support therapy
SpO_2_: oxygen saturation
VD: ventilation device
VM: ventilation mode

## Data Availability

The annotation resources are included in this article. We provide the alarm log scripts [34] as well as the mappings related to airway and medication management interventions [72] on Zenodo. The datasets used and analyzed during this study to conceptualize and generate the alarm annotation resources are stored in the internal network of Charité – Universitätsmedizin Berlin and are not publicly available due to the current data privacy policy. Deidentified data are available from the authors upon reasonable request.

## References

[1] IEC 60601-1-8:2006/AMD2:2020 Amendment 2 - Medical electrical equipment — Part 1-8: General requirements for basic safety and essential performance — Collateral standard: General requirements, tests and guidance for alarm systems in medical electrical equipment and medical electrical systems. IEC (International Electrotechnical Commission).

[2] Görges M, Markewitz BA, Westenskow DR (2009). Improving alarm performance in the medical intensive care unit using delays and clinical context. Anesth Analg.

[3] Tsien CL, Fackler JC (1997). Poor prognosis for existing monitors in the intensive care unit. Crit Care Med.

[4] Siebig S, Kuhls S, Imhoff M, Gather U, Schölmerich J, Wrede CE (2010). Intensive care unit alarms--how many do we need?. Crit Care Med.

[5] Lawless ST (1994). Crying wolf: false alarms in a pediatric intensive care unit. Crit Care Med.

[6] Poncette AS, Mosch L, Spies C, Schmieding M, Schiefenhövel F, Krampe H, Balzer F (2020). Improvements in patient monitoring in the intensive care unit: survey study. J Med Internet Res.

[7] Mosch L, Sümer M, Flint AR, Feufel M, Balzer F, Mörike F, Poncette AS (2024). Alarm management in intensive care: qualitative triangulation study. JMIR Hum Factors.

[8] Sendelbach S, Funk M (2013). Alarm fatigue: a patient safety concern. AACN Adv Crit Care.

[9] Ruskin KJ, Hueske-Kraus D (2015). Alarm fatigue: impacts on patient safety. Curr Opin Anaesthesiol.

[10] Jones K (2014). Alarm fatigue a top patient safety hazard. CMAJ.

[11] Johnson KR, Hagadorn JI, Sink DW (2017). Alarm safety and alarm fatigue. Clin Perinatol.

[12] Poncette AS, Spies C, Mosch L, Schieler M, Weber-Carstens S, Krampe H, Balzer F (2019). Clinical requirements of future patient monitoring in the intensive care unit: qualitative study. JMIR Med Inform.

[13] Wunderlich MM, Amende-Wolf S, Krampe H, Kruppa J, Spies C, Weiß B, Memmert B, Balzer F, Poncette AS (2023). A brief questionnaire for measuring alarm fatigue in nurses and physicians in intensive care units. Sci Rep.

[14] Wunderlich MM, Krampe H, Fuest K, Leicht D, Probst MB, Runge J, Schmid S, Spies C, Weiß B, Balzer F, Poncette AS, CAFQa Study Group Germany (2024). Evaluating the construct validity of the Charité Alarm Fatigue Questionnaire using confirmatory factor analysis. JMIR Hum Factors.

[15] Winters BD, Cvach MM, Bonafide CP, Hu X, Konkani A, O'Connor MF, Rothschild JM, Selby NM, Pelter MM, McLean B, Kane-Gill SL, Society for Critical Care Medicine Alarm and Alert Fatigue Task Force (2018). Technological distractions (part 2): a summary of approaches to manage clinical alarms with intent to reduce alarm fatigue. Crit Care Med.

[16] Chromik J, Klopfenstein SAI, Pfitzner B, Sinno ZC, Arnrich B, Balzer F, Poncette AS (2022). Computational approaches to alleviate alarm fatigue in intensive care medicine: A systematic literature review. Front Digit Health.

[17] Rayo MF, Moffatt-Bruce SD (2015). Alarm system management: evidence-based guidance encouraging direct measurement of informativeness to improve alarm response. BMJ Qual Saf.

[18] Pollard TJ, Johnson AEW, Raffa JD, Celi LA, Mark RG, Badawi O (2018). The eICU Collaborative Research Database, a freely available multi-center database for critical care research. Sci Data.

[19] Faltys M, Zimmermann M, Lyu X, Hüser M, Hyland S, Rätsch G, Merz T (2021). HiRID, a high time-resolution ICU dataset. PhysioNet.

[20] Thoral PJ, Peppink JM, Driessen RH, Sijbrands EJG, Kompanje EJO, Kaplan L, Bailey H, Kesecioglu J, Cecconi M, Churpek M, Clermont G, van der Schaar M, Ercole A, Girbes ARJ, Elbers PWG, Amsterdam University Medical Centers Database (AmsterdamUMCdb) Collaborators and the SCCM/ESICM Joint Data Science Task Force (2021). Sharing ICU patient data responsibly under the Society of Critical Care Medicine/European Society of Intensive Care Medicine joint data science collaboration: The Amsterdam University Medical Centers Database (AmsterdamUMCdb) example. Crit Care Med.

[21] Johnson AEW, Bulgarelli L, Shen L, Gayles A, Shammout A, Horng S, Pollard TJ, Hao S, Moody B, Gow B, Lehman LH, Celi LA, Mark RG (2023). MIMIC-IV, a freely accessible electronic health record dataset. Sci Data.

[22] Johnson A, Bulgarelli L, Pollard T, Gow B, Moody B, Horng S, Celi LA, Mark R (2024). MIMIC-IV (version 3.1). PhysioNet.

[23] Wilken M, Hüske-Kraus D, Röhrig R (2019). Alarm fatigue: using alarm data from a patient data monitoring system on an intensive care unit to improve the alarm management. Stud Health Technol Inform.

[24] Abernethy A (2023). Time for real-world health data to become routine. Nat Med.

[25] Arora A, Alderman JE, Palmer J, Ganapathi S, Laws E, McCradden MD, Oakden-Rayner L, Pfohl SR, Ghassemi M, McKay F, Treanor D, Rostamzadeh N, Mateen B, Gath J, Adebajo AO, Kuku S, Matin R, Heller K, Sapey E, Sebire NJ, Cole-Lewis H, Calvert M, Denniston A, Liu X (2023). The value of standards for health datasets in artificial intelligence-based applications. Nat Med.

[26] Alberto IRI, Alberto NRI, Ghosh AK, Jain B, Jayakumar S, Martinez-Martin N, McCague N, Moukheiber D, Moukheiber L, Moukheiber M, Moukheiber S, Yaghy A, Zhang A, Celi LA (2023). The impact of commercial health datasets on medical research and health-care algorithms. Lancet Digit Health.

[27] de Kok JWTM, de la Hoz MÁA, de Jong Y, Brokke V, Elbers PWG, Thoral P, Castillejo A, Trenor T, Castellano JM, Bronchalo AE, Merz TM, Faltys M, van der Horst ICC, Xu M, Celi LA, van Bussel BCT, Borrat X, Collaborator group (2023). A guide to sharing open healthcare data under the General Data Protection Regulation. Sci Data.

[28] Shillan D, Sterne JAC, Champneys A, Gibbison B (2019). Use of machine learning to analyse routinely collected intensive care unit data: a systematic review. Crit Care.

[29] Xiao C, Choi E, Sun J (2018). Opportunities and challenges in developing deep learning models using electronic health records data: a systematic review. J Am Med Inform Assoc.

[30] Zhang Y, Silvers CT, Randolph AG (2007). Real-time evaluation of patient monitoring algorithms for critical care at the bedside. Annu Int Conf IEEE Eng Med Biol Soc.

[31] Chen L, Dubrawski A, Wang D, Fiterau M, Guillame-Bert M, Bose E, Kaynar AM, Wallace DJ, Guttendorf J, Clermont G, Pinsky MR, Hravnak M (2016). Using supervised machine learning to classify real alerts and artifact in online multisignal vital sign monitoring data. Crit Care Med.

[32] Aboukhalil A, Nielsen L, Saeed M, Mark RG, Clifford GD (2008). Reducing false alarm rates for critical arrhythmias using the arterial blood pressure waveform. J Biomed Inform.

[33] Giesa N, Heeren P, Klopfenstein S, Flint A, Agha-Mir-Salim L, Poncette A, Balzer F, Boie S (2022). MIMIC-IV as a clinical data schema. Stud Health Technol Inform.

[34] Heeren P, Klopfenstein SAI, Poncette AS (2025). Code for "Developing a Scalable Annotation Method for Large Datasets That Enhances Alarms With Actionability Data to Increase Informativeness: Mixed Methods Approach". Zenodo.

[35] Poncette AS, Wunderlich MM, Spies C, Heeren P, Vorderwülbecke G, Salgado E, Kastrup M, Feufel M, Balzer F (2021). Resources for a "Do-it-Yourself Analysis" of the Patient Monitoring Alarm Data from Intensive Care Units. Zenodo.

[36] Hehn J, Mendez D, Hehn J, Mendez D, Brenner W, Broy M (2022). Combining Design Thinking and Software Requirements Engineering to Create Human-Centered Software-Intensive Systems. Design Thinking for Software Engineering. Progress in IS.

[37] Husaria A, Guerreiro S (2020). Requirement Engineering and the Role of Design Thinking. Proceedings of the 22nd International Conference on Enterprise Information Systems (ICEIS 2020).

[38] Thayer RH, Bailin SC, Dorfman M (2000). Software Requirements Engineerings, 2nd Edition.

[39] Laursen LN, Tollestrup C (2017). Design thinking - A paradigm. Proceedings of the 21st International Conference on Engineering Design (ICED 17).

[40] Tran N Design Thinking Playbook for Change Management in K12 Schools. Stanford d.school.

[41] Oliveira M, Zancul E, Fleury AL (2020). Design thinking as an approach for innovation in healthcare: systematic review and research avenues. BMJ Innov.

[42] Altman M, Huang TTK, Breland JY (2018). Design thinking in health care. Prev Chronic Dis.

[43] Vagal A, Wahab SA, Butcher B, Zettel N, Kemper E, Vogel C, Mahoney M (2020). Human-centered design thinking in radiology. J Am Coll Radiol.

[44] Roberts JP, Fisher TR, Trowbridge MJ, Bent C (2016). A design thinking framework for healthcare management and innovation. Healthc (Amst).

[45] Ku B, Lupton E (2022). Health Design Thinking: Creating Products and Services for Better Health (Second Edition).

[46] Krolikowski KA, Bi M, Baggott CM, Khorzad R, Holl JL, Kruser JM (2022). Design thinking to improve healthcare delivery in the intensive care unit: Promise, pitfalls, and lessons learned. J Crit Care.

[47] Wong B (2011). Color blindness. Nat Methods.

[48] Page MJ, McKenzie JE, Bossuyt PM, Boutron I, Hoffmann TC, Mulrow CD, Shamseer L, Tetzlaff JM, Akl EA, Brennan SE, Chou R, Glanville J, Grimshaw JM, Hróbjartsson A, Lalu MM, Li T, Loder EW, Mayo-Wilson E, McDonald S, McGuinness LA, Stewart LA, Thomas J, Tricco AC, Welch VA, Whiting P, Moher D (2021). The PRISMA 2020 statement: An updated guideline for reporting systematic reviews. PLoS Med.

[49] (2006). IEC 60601-1-8:2006 - Medical electrical equipment — Part 1-8: General requirements for basic safety and essential performance — Collateral standard: General requirements, tests and guidance for alarm systems in medical electrical equipment and medical electrical systems. IEC (International Electrotechnical Commission).

[50] (2012). IEC 60601-1-8:2006/Amd 1:2012 Amendment 1 - Medical electrical equipment — Part 1-8: General requirements for basic safety and essential performance — Collateral standard: General requirements, tests and guidance for alarm systems in medical electrical equipment and medical electrical systems. IEC (International Electrotechnical Commission).

[51] (2019). ISO 19223:2019 Lung ventilators and related equipment — Vocabulary and semantics. ISO (International Organization for Standardization).

[52] Heeren P, Klopfenstein SAI, Prendke M, Balzer F, Poncette AS (2023). What medication actions follow patient monitoring alarms in intensive care units? A retrospective analysis. German Medical Science.

[53] Siebig S, Kuhls S, Imhoff M, Langgartner J, Reng M, Schölmerich J, Gather U, Wrede CE (2010). Collection of annotated data in a clinical validation study for alarm algorithms in intensive care--a methodologic framework. J Crit Care.

[54] Schmid F, Goepfert MS, Kuhnt D, Eichhorn V, Diedrichs S, Reichenspurner H, Goetz AE, Reuter DA (2011). The wolf is crying in the operating room: patient monitor and anesthesia workstation alarming patterns during cardiac surgery. Anesth Analg.

[55] Borowski M, Siebig S, Wrede C, Imhoff M (2011). Reducing false alarms of intensive care online-monitoring systems: an evaluation of two signal extraction algorithms. Comput Math Methods Med.

[56] Scalzo F, Liebeskind D, Hu X (2013). Reducing false intracranial pressure alarms using morphological waveform features. IEEE Trans Biomed Eng.

[57] Scalzo F, Hu X (2013). Semi-supervised detection of intracranial pressure alarms using waveform dynamics. Physiol Meas.

[58] Inokuchi R, Sato H, Nanjo Y, Echigo M, Tanaka A, Ishii T, Matsubara T, Doi K, Gunshin M, Hiruma T, Nakamura K, Shinohara K, Kitsuta Y, Nakajima S, Umezu M, Yahagi N (2013). The proportion of clinically relevant alarms decreases as patient clinical severity decreases in intensive care units: a pilot study. BMJ Open.

[59] Drew BJ, Harris P, Zègre-Hemsey JK, Mammone T, Schindler D, Salas-Boni R, Bai Y, Tinoco A, Ding Q, Hu X (2014). Insights into the problem of alarm fatigue with physiologic monitor devices: a comprehensive observational study of consecutive intensive care unit patients. PLoS One.

[60] Clifford G, Silva I, Moody B, Li Q, Kella D, Shahin A, Kooistra T, Perry D, Mark RG (2015). The PhysioNet/Computing in Cardiology Challenge 2015: Reducing false arrhythmia alarms in the ICU. Comput Cardiol (2010).

[61] Clifford GD, Silva I, Moody B, Li Q, Kella D, Chahin A, Kooistra T, Perry D, Mark RG (2016). False alarm reduction in critical care. Physiol Meas.

[62] Zong W, Nielsen L, Gross B, Brea J, Frassica J (2016). A practical algorithm to reduce false critical ECG alarms using arterial blood pressure and/or photoplethysmogram waveforms. Physiol Meas.

[63] Hravnak M, Chen L, Dubrawski A, Bose E, Clermont G, Pinsky MR (2016). Real alerts and artifact classification in archived multi-signal vital sign monitoring data: implications for mining big data. J Clin Monit Comput.

[64] Schmid F, Goepfert MS, Franz F, Laule D, Reiter B, Goetz AE, Reuter DA (2017). Reduction of clinically irrelevant alarms in patient monitoring by adaptive time delays. J Clin Monit Comput.

[65] Harris PR, Zègre-Hemsey JK, Schindler D, Bai Y, Pelter MM, Hu X (2017). Patient characteristics associated with false arrhythmia alarms in intensive care. Ther Clin Risk Manag.

[66] Nizami S, Basharat A, Shoukat A, Hameed U, Raza S, Bekele A (2018). CEA: Clinical Event Annotator mHealth Application for Real-time Patient Monitoring. https://ieeexplore.ieee.org/document/8512898.

[67] Assis AP, Oliveira FT, Camerini FG, Silva RCLD, Moraes CM (2019). Individualized parameterization of multiparametric monitors alarms in infarcted patients. Rev Bras Enferm.

[68] Suba S, Sandoval CP, Hu X, Pelter MM (2019). ECG Monitoring during End of Life Care: Implications on Alarm Fatigue. MTI.

[69] Fernandes C, Miles S, Lucena CJP (2020). Detecting false alarms by analyzing alarm-context information: algorithm development and validation. JMIR Med Inform.

[70] Au-Yeung WM, Sevakula RK, Sahani AK, Kassab M, Boyer R, Isselbacher EM, Armoundas AA (2021). Real-time machine learning-based intensive care unit alarm classification without prior knowledge of the underlying rhythm. Eur Heart J Digit Health.

[71] Flint AR, Klopfenstein SAI, Heeren P, Balzer F, Poncette AS (2022). Utilizing intensive care alarms for machine learning. Stud Health Technol Inform.

[72] Klopfenstein SAI, Flint AR, Heeren P, Prendke M, Chaoui A, Ocker T, Chromik J, Arnrich B, Balzer F, Poncette AS (2024). Mappings for "Developing a Scalable Annotation Method for Large Datasets That Enhances Alarms With Actionability Data to Increase Informativeness: Mixed Methods Approach". Zenodo.

[73] Harutyunyan H, Khachatrian H, Kale DC, Ver Steeg G, Galstyan A (2019). Multitask learning and benchmarking with clinical time series data. Sci Data.

[74] Johnson AEW, Pollard TJ, Shen L, Lehman LH, Feng M, Ghassemi M, Moody B, Szolovits P, Celi LA, Mark RG (2016). MIMIC-III, a freely accessible critical care database. Sci Data.

[75] National Patient Safety Goals® Effective January 2024 for the Hospital Program. The Joint Commission.

[76] Ruppel H, Pohl E, Rodriguez-Paras C, Froh E, Perry K, McNamara M, Muthu N, Ferro D, Rasooly I, Bonafide CP (2023). Clinician perspectives on specifications for metrics to inform pediatric alarm management. Biomed Instrum Technol.

[77] Wac M, Santos-Rodriguez R, McWilliams C, Bourdeaux C (2023). CATS: Cloud-native time-series data annotation tool for intensive care. SoftwareX.

[78] Reiter N, Willand M, Gius E (2019). A shared task for the digital humanities chapter 1: Introduction to annotation, narrative levels and shared tasks. Journal of Cultural Analytics.

[79] Santorini B (1990). Part-of-Speech Tagging Guidelines for the Penn Treebank Project (3rd Revision). LDC Catalog.

[80] Tseng T, Stent A, Maida D (2020). Best Practices for Managing Data Annotation Projects. arXiv.

[81] Chang AM (2018). Lean Impact: How to Innovate for Radically Greater Social Good.

[82] Chromik J, Pfitzner B, Ihde N, Michaelis M, Schmidt D, Klopfenstein SAI, Poncette AS, Balzer F, Arnrich B (2022). Extracting Alarm Events from the MIMIC-III Clinical Database. Proceedings of the 15th International Joint Conference on Biomedical Engineering Systems and Technologies.

[83] Smit JM, Krijthe JH, van Bommel J, Causal Inference for ICU Collaborators (2023). The future of artificial intelligence in intensive care: moving from predictive to actionable AI. Intensive Care Med.

[84] Yu L, Halalau A, Dalal B, Abbas AE, Ivascu F, Amin M, Nair GB (2021). Machine learning methods to predict mechanical ventilation and mortality in patients with COVID-19. PLoS One.

[85] Muroi C, Meier S, De Luca V, Mack DJ, Strässle C, Schwab P, Karlen W, Keller E (2020). Automated false alarm reduction in a real-life intensive care setting using motion detection. Neurocrit Care.

[86] Bai Y, Do DH, Harris PRE, Schindler D, Boyle NG, Drew BJ, Hu X (2015). Integrating monitor alarms with laboratory test results to enhance patient deterioration prediction. J Biomed Inform.

[87] Bai Y, Do D, Ding Q, Palacios JA, Shahriari Y, Pelter MM, Boyle N, Fidler R, Hu X (2017). Is the sequence of SuperAlarm triggers more predictive than sequence of the currently utilized patient monitor alarms?. IEEE Trans Biomed Eng.

[88] Hu X, Sapo M, Nenov V, Barry T, Kim S, Do DH, Boyle N, Martin N (2012). Predictive combinations of monitor alarms preceding in-hospital code blue events. J Biomed Inform.

[89] Segall N, Borbolla D, Del Fiol G, Waller R, Reese T, Nesbitt P (2017). Trend Displays to Support Critical Care: A Systematic Review.

[90] Kamaleswaran R, Collins C, James A, McGregor C (2016). PhysioEx: visual analysis of physiological event streams. Computer Graphics Forum.

[91] Kamaleswaran R, James A, Collins C, McGregor C (2016). CoRAD: Visual Analytics for Cohort Analysis.

[92] Edworthy JR, Schlesinger JJ, McNeer RR, Kristensen MS, Bennett CL (2017). Classifying alarms: seeking durability, credibility, consistency, and simplicity. Biomed Instrum Technol.

[93] INALO - Intelligenter Alarmoptimierer für die Intensivstation. Bundesministerium für Bildung und Forschung.

