# Supplementary material for: Developing a Scalable Annotation Method for Large Datasets That Enhances Alarms With Actionability Data to Increase Informativeness: Mixed Methods Approach
Source: J Med Internet Res. 2025 May 5;27:e65961. doi: 10.2196/65961 (PMC12089878; doi:10.2196/65961)
Supplement: Multimedia Appendix 1 [file jmir_v27i1e65961_app1.docx]

## **Multimedia Appendix 1.** Demographics

## *This is a Multimedia Appendix to a full manuscript published in the J Med Internet Res. For full copyright and citation information see* [*http://dx.doi.org/10.2196/65961*](http://dx.doi.org/10.2196/65961)

**Table S1.** Counts of distinct cases and patients per intensive care unit ward.

| Intensive care unit ward | Case count, n | Patient count^a^, n |
| --- | --- | --- |
| M101I | 1434 | 1385 |
| M102I | 888 | 839 |
| M103I | 693 | 678 |
| M203AI | 123 | 123 |
| M203BI | 265 | 264 |
| M204AI | 16 | 16 |
| M204BI | 67 | 67 |
| S44I | 1162 | 1119 |
| W1I | 923 | 896 |
| W9I | 534 | 526 |
| WAC-S21I | 195 | 188 |
| WAN-S14I | 1101 | 1071 |
| WAN-S8I | 631 | 626 |

^a^Note that the patient count in this table is higher than that in Table S2 because patients transferred between different intensive care units are counted multiple times.

**Table S2.** Age and gender distribution of intensive care unit patients.

| Age decade | Female patients, n | Male patients, n |
| --- | --- | --- |
| 10-20 | 54 | 47 |
| 20-30 | 172 | 147 |
| 30-40 | 185 | 288 |
| 40-50 | 294 | 428 |
| 50-60 | 477 | 862 |
| 60-70 | 632 | 1015 |
| 70-80 | 718 | 915 |
| 80-90 | 405 | 421 |
| 90-100 | 63 | 40 |
