# Supplementary material for: Developing a Scalable Annotation Method for Large Datasets That Enhances Alarms With Actionability Data to Increase Informativeness: Mixed Methods Approach
Source: J Med Internet Res. 2025 May 5;27:e65961. doi: 10.2196/65961 (PMC12089878; doi:10.2196/65961)
Supplement: Multimedia Appendix 3 [file jmir_v27i1e65961_app3.docx]

## **Multimedia Appendix 3.** Questions asked to experts during one-on-one meetings and workshops, and challenges identified through exploratory analyses of the data sources, structures, and entries from our institution.

## *This is a Multimedia Appendix to a full manuscript published in the J Med Internet Res. For full copyright and citation information see* [*http://dx.doi.org/10.2196/65961*](http://dx.doi.org/10.2196/65961)

**Table S1.** Overview of questions that guided one-on-one meetings and workshops with experts.

| Questions asked to medical experts | Questions asked to the information technology expert |
| --- | --- |
| We want to design a system that helps to recognize which alarms might require an intervention and which do not. To achieve this, we want to create a dataset of alarms labelled based on their actionability   - How would you approach this task? - What data would you use? | Can you describe the governance processes around ICU devices and systems (including the PDMS)? |
| Regarding documentation in your daily work:   - Do you document (every) intervention that you perform? - How timely do you document interventions after you perform them? - Are there any interventions that you are more likely to document, and others that you are less likely to document? - Are there any interventions that you are more likely to document in a timely manner? | Does all produced data get stored?   - If so, where? - If not, who decides what data should be stored? |
| Regarding alarm management:   - Do you react to every alarm? - Do you take actions to reduce the number of alarms or get alarmed less often? - Do you document your reaction to alarms? | Are any standard operating procedures currently used related to data storage and storage frequency? |
| If we were to focus on specific alarm types to start with, which would you recommend? | Regarding the automatic transfer of data from devices to the PDMS:   - For devices where values are automatically transmitted to the PDMS, how big is the delay between measurement and storage? - Can medication pumps automatically transfer their parameters? If so, why is it not currently used? - Are there any plans to automatically transfer alarm data, such as set limits or occurring alarms, to the PDMS? |
| We grouped the different alarm types by their associated PAC. Please comment, correct and validate the list. | Regarding the subset of data the PDMS displays:   - Who decides what data is displayed? - Who decides whether data is only displayed if a change occurred or at regular defined intervals? - Why do some variables have different application timestamps (“charttime” and “storetime”)? |
| Regarding different alarm types:   - Could you name frequent causes that lead to alarm signals? - Could you list some potential interventions that you would perform for these causes? - Could you rank these interventions by frequency based on your experience? - Are there any interventions that you would only perform in case of co-occurring alarms? - Are there any interventions that you would only perform after an alarm train? | Regarding the individual policies of each ICU which data to display and how often (e.g., at regular defined intervals, whenever there is a change, or both):   - Is it possible to get an overview of these settings? - Are displayed numerical values the exact values or aggregated values (e.g., mean, median)? |
| If we were to focus on specific interventions to start with, which would you recommend? | Regarding currently used documentation control mechanisms:   - How can some vital signs produce negative values? Are there any mechanisms to address this issue? - Are there any mechanisms that control what can be documented in specific fields, e.g., enforcement of ICD10 for diagnoses? |
| Regarding the partially structured ventilation data in the PDMS:   - Which information would you use to determine if an AD was placed, replaced or removed? - Which information would you use to determine the invasiveness of the respiratory management? - Are there any resources at our institution that summarize the different VDs, their associated VMs and the correct AD to use? - Do you know the norm ISO19223 and its categorization system? - We queried all AD, VD, and VM information from our PDMS and created mappings. Could you comment, correct, and validate our list? | Regarding the partially structured ventilation data in the PDMS:   - Why is it possible to add additional AD, VD and VM as free text? - Why are there two variables in the PDMS that store information on VD and VM? One variable stores automatically transmitted data, the other is used for manual documentation. |
| Regarding medication management:   - Based on frequent underlying pathologies and etiologies leading to an alarm condition, could you name substances for each alarm type that you might administer or reduce, and specify what you would do (administration or reduction)? - Which substances do you often manage as a reaction to an alarm? - How timely do you document these medication interventions? - Do you also document single boli? - Some medication products are already planned to be administered at defined time intervals/time points or if necessary. Existing short cuts in the PDMS enable users to declare these administrations as given. Do you adjust the timestamp if you gave them as a reaction to an alarm? - Based on each PAC, we performed mappings including the DrugIDs used in our institution, substance names, routes and techniques of administration. Could you comment, correct and validate our list | Regarding the lists of medication products our institution currently manages, that includes information on substances, generic names, application form and route, dosage, etc.:   - How did you develop the structure? - How do you assign DrugIDs to single medication products? - Do you also assign DrugIDs to mixtures? - Can you talk about the management processes of these lists? - Are there versioning processes involved? |
| Do you have any further suggestions on how to create our labelled dataset, or what we should take into account, including data points we should consider or challenges that we might not considered previously? | |

**Table S2.** Results of the exploratory analysis of data sources, structures and entries.

| Findings | Examples and/or Explanations | Challenges |
| --- | --- | --- |
| Nested database structure | Example: Complex, nested medication model in the PDMS | Identification, extraction and preprocessing of necessary variables can be complex and time-intensive |
| Duplicate information between systems | Example: Lab results are stored in the PDMS and HIS | De-duplication challenging due to the large amount of data; it might be useful to decide on one data source to extract variables that are known to be duplicated in different systems |
| Different documentation types: manually vs. automatically | Data from some devices, such as medication pumps, despite having a connection to the PDMS, need to be entered manually for legal reasons | Manual documentation can be error-prone and might not occur timely after an event. |
| Different data formats and structures | Unstructured and structured; string (predefined or free text or both), integer, date, codes from terminologies, etc. | Complex data preprocessing and mappings are necessary to be able to use and compare the data |
| Different storage frequency and data accuracy of same features across ICUs | Every ICU can set the storage and accuracy preferences in its instance of the PDMS – the settings cannot be assessed centrally.  Example 1: A parameter might be saved every time it changes, on a regular basis (for instance every 5 minutes), or both.  Example 2: A parameter might be saved as an exact value or as an average of the values measured in a set time period.  Example 3: Timestamps might be rounded up or down (e.g., to the nearest 5 minutes). | Difficult to evaluate if data reflect the reality (did an event happen at point in time X?) |
| Different timestamps might be documented for a single variable | Two timestamps can be stored for a set of variables: “storetime” is the timestamp when the variable has been entered or updated (either manually or automatically), while “charttime” is the timestamp representing the time when the event happened. We noticed that the “storetime” might be logged before the “charttime” for automatically transmitted variables, but not for manually documented ones. On the other hand, the “storetime” might be later than the “charttime”, e.g., when personnel document interventions retrospectively. | Decision which timestamp should be used in which situation can be complex and should be based on the evaluation of different scenarios.  Timestamps might only be used as approximate values of events’ occurrence. |
| Systems and devices might use different time zones | Example: Coordinated Universal Time, and local summer and winter time. | All timestamps need to be converted to a common time zone before being used for time series analyses. |
| PDMS does not perform medical plausibility checks | Example 1: The PDMS cannot assess based on the relevant information if the respiratory support therapy is invasive or non-invasive – as defined in ISO19223 3.1.3^46^.  Example 2: The PDMS does not indicate – except for lab parameters – if the values stored are in the physiological range, or even if they are plausible and compatible with life.  Example 3: We noticed that some ventilation devices still transmitted values for set ventilation parameters to the PDMS while their mode was standby. | Complex mappings and data processing are necessary but also time- and resource-intensive. Device modes matter: The meaning of values transmitted during standby mode differs from the meaning in other modes. When a device is in standby, setting values can still be automatically transmitted. However e.g., when a ventilator is in standby, its functions are suspended: the patient is not ventilated at this moment in time (at least using this specific ventilator).  Therefore, plausible values should be defined and documented to clean the dataset. |
| Alarm logs are stored locally and temporarily without integration in the PDMS or HIS | Philips IntelliVue central station stores the patient alarm logs locally for 90 days maximum. Alarm logs can be collected using USB storage devices. | Alarm data can get lost if not collected on-site by personnel within 90 days. |
| Alarm logs need extensive processing | The alarm logs are comma-separated value files (csv files) and contain the bed name, a timestamp when the alarm was entered in the log, and a string with alarm information (alarm types, criticality, timestamps such as start, end, pause, measured values of the monitored vital signs and thresholds that are exceeded or undershot). This information is extracted using Regular Expression. Additionally alarms do not have identifying identifiers nor include patient identifying information. | Without identifying identifiers, alarm start and end can only be correlated by sorting the alarm log entries chronologically in an error-prone best effort.  To be used in combination with patient data, alarm logs need to be matched to the respective ICU patients using bed name and timestamp information from the PDMS and HIS. |
