# Supplementary material for: Developing a Scalable Annotation Method for Large Datasets That Enhances Alarms With Actionability Data to Increase Informativeness: Mixed Methods Approach
Source: J Med Internet Res. 2025 May 5;27:e65961. doi: 10.2196/65961 (PMC12089878; doi:10.2196/65961)

## **Multimedia Appendix 4.** Proposed annotation output structure and examples illustrating the preprocessing steps and annotation algorithm.

## *This is a Multimedia Appendix to a full manuscript published in the J Med Internet Res. For full copyright and citation information see* [*http://dx.doi.org/10.2196/65961*](http://dx.doi.org/10.2196/65961)

**Table S1.** Annotation output structure for respiratory management interventions.

| Column Name | Alarmlog_id | Annotation_rule | Actionable_time | Pre_value | Pre_Mapping | Pre_Invasiveness_Degree | Post_value | Post_Mapping | Post_Invasiveness_Degree | Alarm_indication |
| --- | --- | --- | --- | --- | --- | --- | --- | --- | --- | --- |
| Data Type | TEXT | TEXT | DATETIME | TEXT | TEXT | TEXT | TEXT | TEXT |  | TEXT |
| Description | Alarm log identifier | Rule that made the alarm actionable | Time of the intervention leading to the alarm being actionable | Last value or value at the time of the alarm used for comparison | Mapping to category (AD or RST) when relevant, otherwise N/A | Last value or value at the time of the alarm | (Maximal) value in the specified post-alarm time window used for comparison | Mapping to category (AD or RST) when relevant, otherwise N/A | Maximal value in the specified post-alarm time window | Physiological Alarm Condition |
| Example | 3gfjbskhfza7skdbed63n3o76s3ohf76 | Insertion or change of airway device | 2019-10-10 13:15:00 | 1 | Blank / No AD | 3 | 4 | Oxygen Mask | 2 | spo2_low |

**Table S2**. Annotation output structure for medication management interventions.

| Column Name | Alarmlog_id | Annotation_rule | Actionable_time | DrugID | Pre_value | Post_value | Alarm_indication |  |
| --- | --- | --- | --- | --- | --- | --- | --- | --- |
| Data Type | TEXT | TEXT | DATETIME | INT | DOUBLE | DOUBLE | TEXT |  |
| Description | Alarm log identifier | Rule that made the alarm actionable | Time of the intervention leading to the alarm being actionable | DrugID of the medication leading to the alarm being actionable | Dose per minute related to the administration at the time of the alarm, and used for comparison. In case of a bolus: “n/a”. | Dose (for continuous administration: per minute) related to the (new) administration post-alarm | Physiological Alarm Condition |  |
| Example 1 | | fae3680521c3eeacafe30dbf3388e01e | Bolus | 2020-08-10 17:55:51 | 214 | n/a | 100.0 | bp_low |
| Example 2 | af89e962dce22a5ff206cb0539846dfb | Increase | 2020-08-10 17:57:00 | 236 | 33.6 | 68.57 | hf_low |  |

**Table S3.** Airway device (AD) categories, mapping results (number) and examples from the patient data management system (PDMS).

| AD Categories | | | Number of ADs mapped to category | Examples from PDMS | |
| --- | --- | --- | --- | --- | --- |
| AD_LEVEL | AD_CATEGORY_ID | AD_CATEGORY_NAME |  | AD_ENTRY _ID | AD_ENTRY_NAME |
| 1 | 1 | Blank / No AD | 3 | 1 | “ “ |
| 2 | 10 | Oropharyngeal Airway | 4 | 135 | “Güdel” |
| 2 | 11 | Nasopharyngeal Airway | 15 | 743 | “Wendel” |
| 3 | 2 | Oxygen Nasal Cannula | 45 | 303 | “O2-Nasenbrille” |
| 4 | 4 | Oxygen Mask | 24 | 301 | “O2-Maske” |
| 5 | 5 | Nasal CPAP Mask | 68 | 5 | “Air Fit N30i” |
| 6 | 6 | Full Face CPAP Mask | 108 | 411 | “Resmed AirFit F30” |
| 7 | 3 | High Flow Nasal Cannula | 84 | 141 | “HFNC-Brille” |
| 8 | 8 | Laryngeal Mask | 44 | 202 | “LMA i-gel” |
| 9 | 7 | Tracheal Cannula *(including Speech Valve or Cannula)* | 460 | 526 | “TK Rüsch” |
| 9 | 9 | Endotracheal Tube | 251 | 225 | “Magill Tubus” |
| 0 | 12 | Bag Valve Mask | 2 | 755 | “Ambubeutel” |
| 0 | 13 | Inhaler or Breathing Exerciser | 9 | 172 | “inhalette” |
| 0 | 14 | (Tracheostoma) Placeholder | 8 | 338 | “Platzhalter” |
| 0 | 15 | Temperature / Esophageal probe | 2 | 330 | “Ösophagustemperatursonde” |
| 0 | 16 | Nasal Stents | 3 | 35 | “Choanalröhrchen” |
| 0 | 17 | Palate Plate | 4 | 128 | “Gaumenplatte” |
| 0 | 18 | Documentation Error | 1 | 739 | “versehent. DOPPLUNG” |

**Table S4.** Structure of the medication mapping with numbers (N) of active ingredients and examples from the database. Note: The column “active ingredients (N)” is not part of the original mapping table. Abbreviations: BP: blood pressure; HR: heartrate.

| Medication interventions | PAC | Active ingredients (N) | Examples | | | |
| --- | --- | --- | --- | --- | --- | --- |
|  |  |  | Active ingredient | DrugID | Route of administration | Technique of administration |
| Administration or increase in dosage | SpO2_low | 18 | Salbutamol | 149 | Inhalative, endobronchial or intratracheal | Bolus |
|  | HR_low | 4 | Epinephrine | 236 | Intravenous – continuous | Continuous |
|  | HR_high | 16 | Amiodarone | 5784 | Intravenous – bolus | Bolus |
|  | BP_low | 8 | Norepinephrine | 214 | Intravenous – bolus | Bolus |
|  | BP_high | 7 | Glyceryl trinitrate | 1717 | Sublingual | Bolus |
| Administration stopped or reduction of dosage | HR_low | 6 | Esmolol | 3922 | Intravenous – continuous | Continuous |
|  | HR_high | 2 | Dobutamine | 234 | Intravenous – continuous | Continuous |
|  | BP_low | 5 | Clonidine | 5590 | Intravenous – continuous | Continuous |
|  | BP_high | 4 | Vasopressin | 259 | Intravenous – continuous | Continuous |

**Figure S1.** Medication “Bolus” annotation rule with example data and annotation result
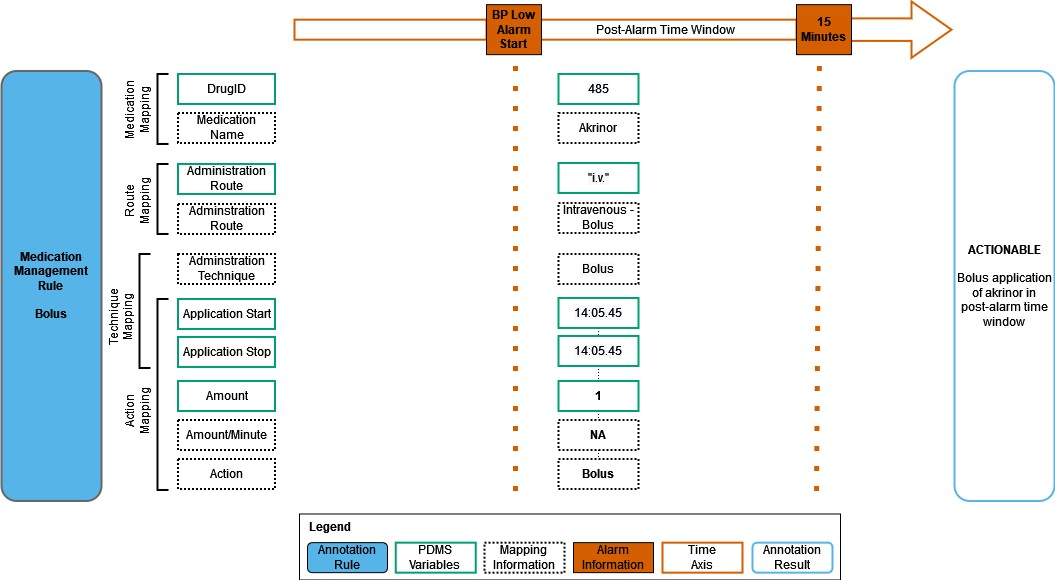


**Figure S2.** Medication “Start” annotation rule with example data and annotation result.


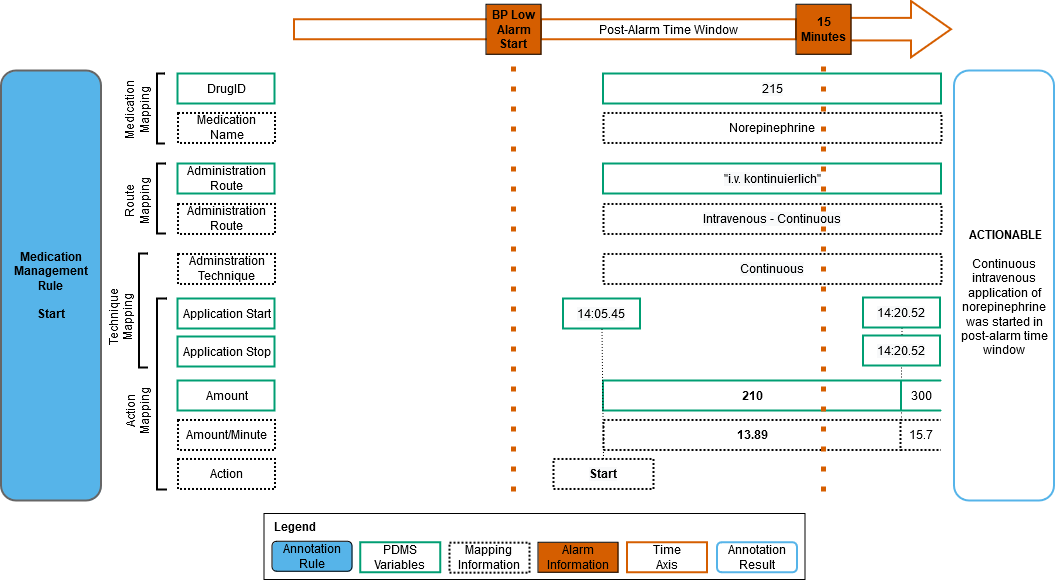


**Figure S3.** Medication “Stop” annotation rule with example data and annotation result


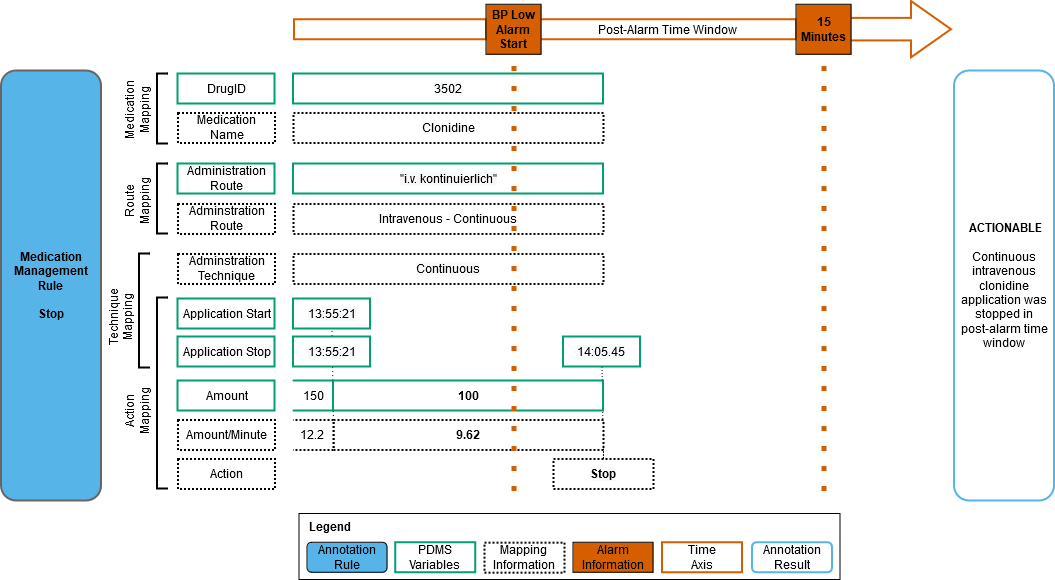


**Figure S4.** Medication “Decrease” annotation rule with example data and annotation result


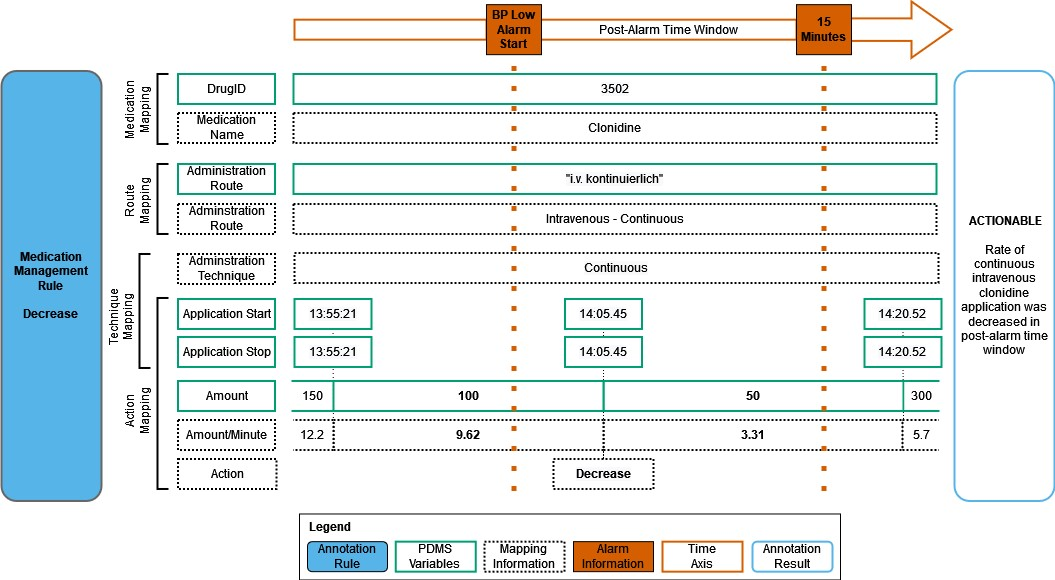

Supplement: Multimedia Appendix 4 [file jmir_v27i1e65961_app4.docx]
